# Biotechnology approach to determination of genetic and epigenetic control in cells

**DOI:** 10.1186/1477-3155-2-11

**Published:** 2004-11-22

**Authors:** Kenji Yasuda

**Affiliations:** 1 Department of Life Sciences, Graduate school of Arts and Sciences, University of Tokyo, 3-8-1 Komaba, Meguro, Tokyo 153-8902 JAPAN

## Abstract

A series of studies aimed at developing methods and systems for analyzing epigenetic information in cells are presented. The role of the epigenetic information of cells, which is complementary to their genetic information, was inferred by comparing the predictions of genetic information with the cell behaviour observed under conditions chosen to reveal adaptation processes and community effects. Analysis of epigenetic information was developed starting from the twin complementary viewpoints of cells regulation as an 'algebraic' system (emphasis on the temporal aspect) and as a 'geometric' system (emphasis on the spatial aspect). The knowlege acquired from this study will lead to the use of cells for fully controlled practical applications like cell-based drug screening and the regeneration of organs.

## Review

### 1. General background

**Figure 1:**
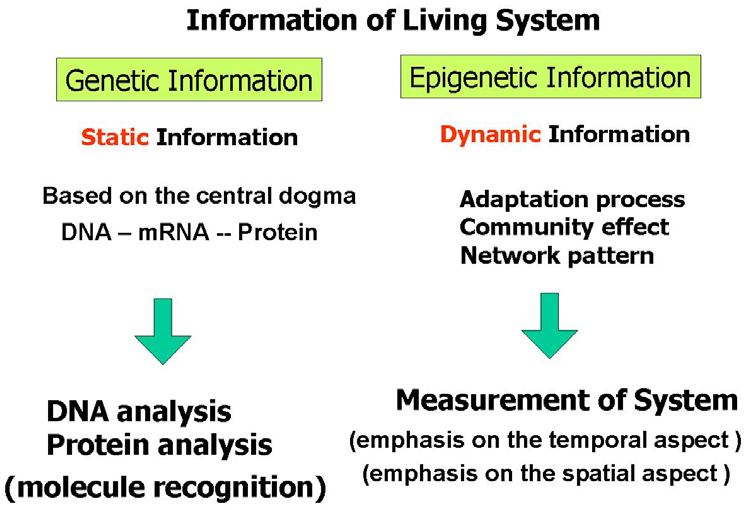
Epigenetic information: complementary to genetic information.

Knowledge about living organisms increased dramatically during the 20th century and has produced the modern disciplines of genomics and proteomics. Despite these advances, however, there remains the great challenge of learning how the different living components of the cell are integrated and regulated. As we move into the post-genomic period, the complementarity of genomics and proteomics will become apparent and the connections between them will be exploited. However, neither genomics nor proteomics alone can provide the knowledge needed to interconnect the molecular events in living cells. The cells in a group are individual entities, and differences arise even among cells with identical genetic information that have grown under the same conditions. These cells respond to perturbations differently. Why and how do these differences arise? Cells are the minimum units containing both genetic and epigenetic information which are used in response to environmental conditions such as interactions between neighbouring cells and of changes in extracellular conditions. To understand the rules underlying the possible differences occurring in cells, we need to develop methods for simultaneously evaluating both the genetic information and the epigenetic information (Fig. 1). In other words, if we are to understand adaptation processes, community effects, and the meaning of network patterns of cells, we need to analyze the epigenetic information in cells. Thus we have started a project focusing on developing a system that can be used to evaluate the epigenetic information of cells by observing specific cells and their interactions continuously under controlled conditions. The importance of the understanding of epigenetic information will become apparent in cell-based biological and medical fields like cell-based drug screening and the regeneration of organs from stem cells, fields in which phenomena cannot be interpreted without taking epigenetic factors into account.

**Figure 2:**
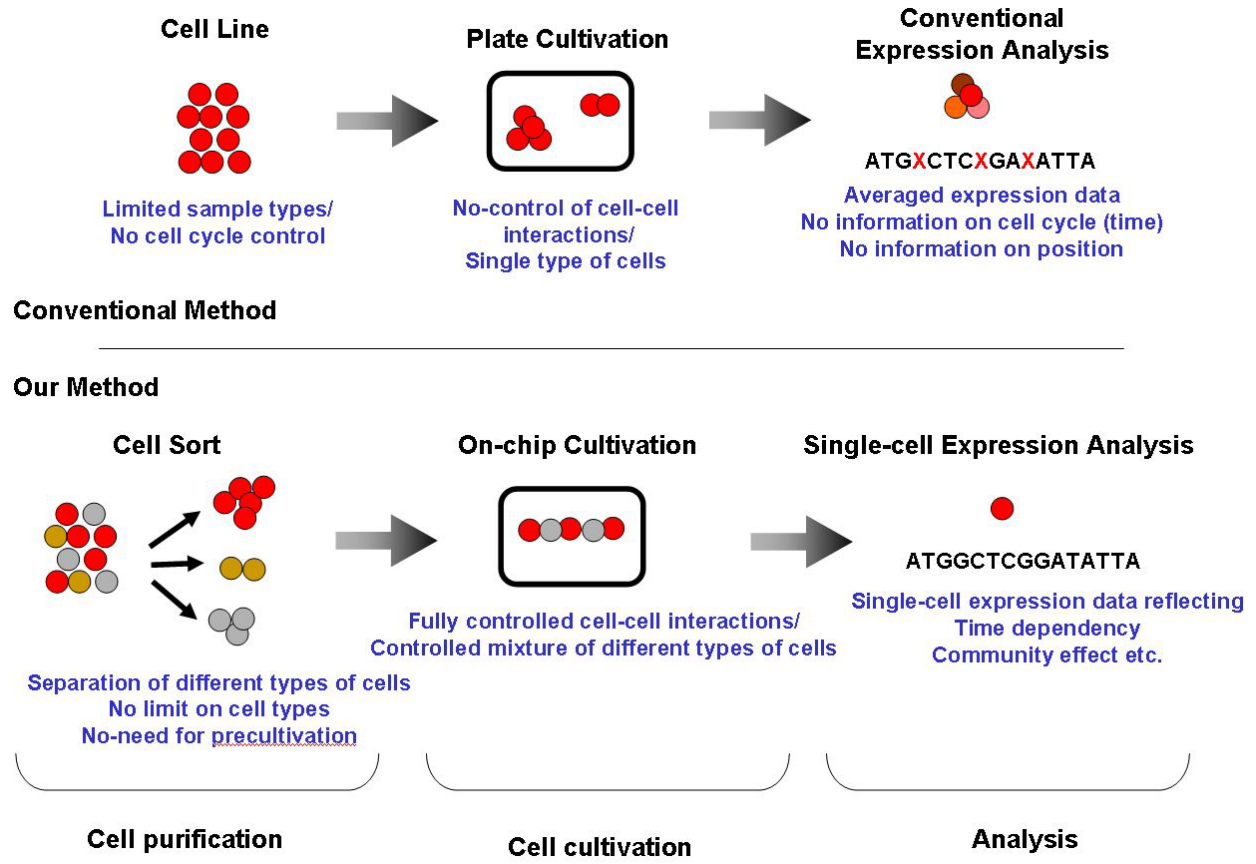
Our strategy: on-chip single-cell-based analysis.

In 1999 the author moved to the Univ. of Tokyo and began his research on the "determination of genetic and epigenetic cellular control processes". To understand the meaning of the genetic variability and the epigenetic correlation of cells, we have developed the on-chip single-cell-based microcultivation method. As shown in Fig. 2, the strategy consists of a three step process. First we purify cells from tissue individually in a nondestructive manner. [1] Then we cultivate the cells and observe them under fully controlled conditions (*e.g*., cell population, network pattern, or nutrient conditions) by using the on-chip single-cell cultivation chip [2-10] or by using an on-chip agarose microchamber system [11-14]. Finally, we do a single-cell-based expression analysis using the photothermal denaturation method and a single-molecule level analysis [15]. In this way, we can control the spatial distribution and interactions of cells.

### 2. Aim of the project

The aim of our project is to develop methods and systems for analyzing the epigenetic information in cells. The project is based on the idea that, although genetic information makes a network of biochemical reactions, the history of the network as a parallel-processing recurrent network was ultimately determined by the environmental conditions of cells, which we call epigenetic information. As described above, if we are to understand the events in living systems at the cellular level, we need to keep in mind that epigenetic information is complementary to genetic information.

The advantage of this approach is that it bypasses the complexity of underlying physicochemical reactions which are not always completely understood and for which most of the necessary variables cannot be measured. Moreover, this approach shifts the view of cell regulatory processes from the basic chemical ground to the paradigm of a cell as an information-processing unit working as an intelligent machine capable of adaptation to changing environmental and internal conditions. It is an alternative representation of the cell and can bring new insight into cellular processes. Moreover, models derived from such a viewpoint can directly help in the more traditional biochemical and molecular biological analyses of cell control.

**Figure 3:**
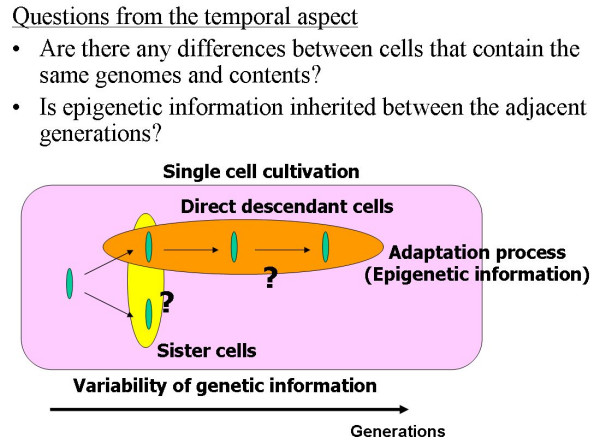
Aim of our project (1): temporal aspect.

**Figure 4:**
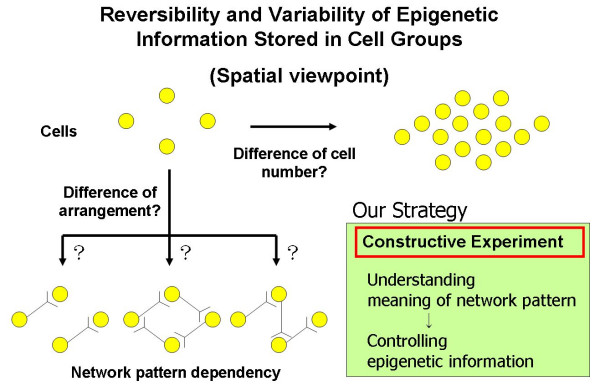
Aim of our project (2): spatial aspect.

The basic part of the project is the development of on-chip single-cell-based cultivation and analysis systems for monitoring the dynamic processes in the cell. In addition we have employed these systems to examine a number of other processes eg; the variability of cells having the same genetic information, the inheritance of non-genetic information between adjacent generations of cells, the cellular adaptation processes caused by environmental change, the community effect of cells and network pattern formation in cell groups (Figs. 3 and 4). After making extensive experimental observations, we can understand the meaning of epigenetic information in the modeling of more complex signaling cascades. This field has been largely monopolized by physico-chemical models, which provide a good standard for the comparison, evaluation, and development of our approach. The ultimate aim of our project is to provide a comprehensive understanding of living systems as the products of both genetic information and epigenetic information.

### 3-1. Single-cell cultivation chip system [2-10]

**Figure 5:**
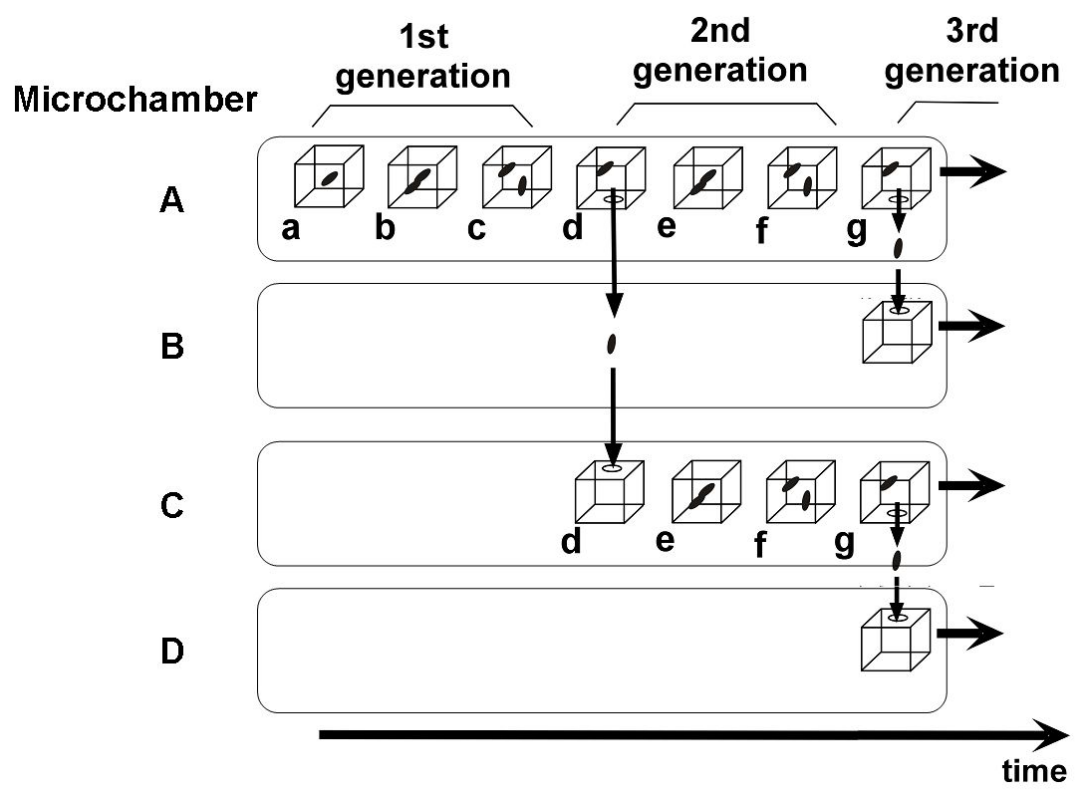
Single-cell cultivation in microchambers for measuring the variability of genetic information.

**Figure 6:**
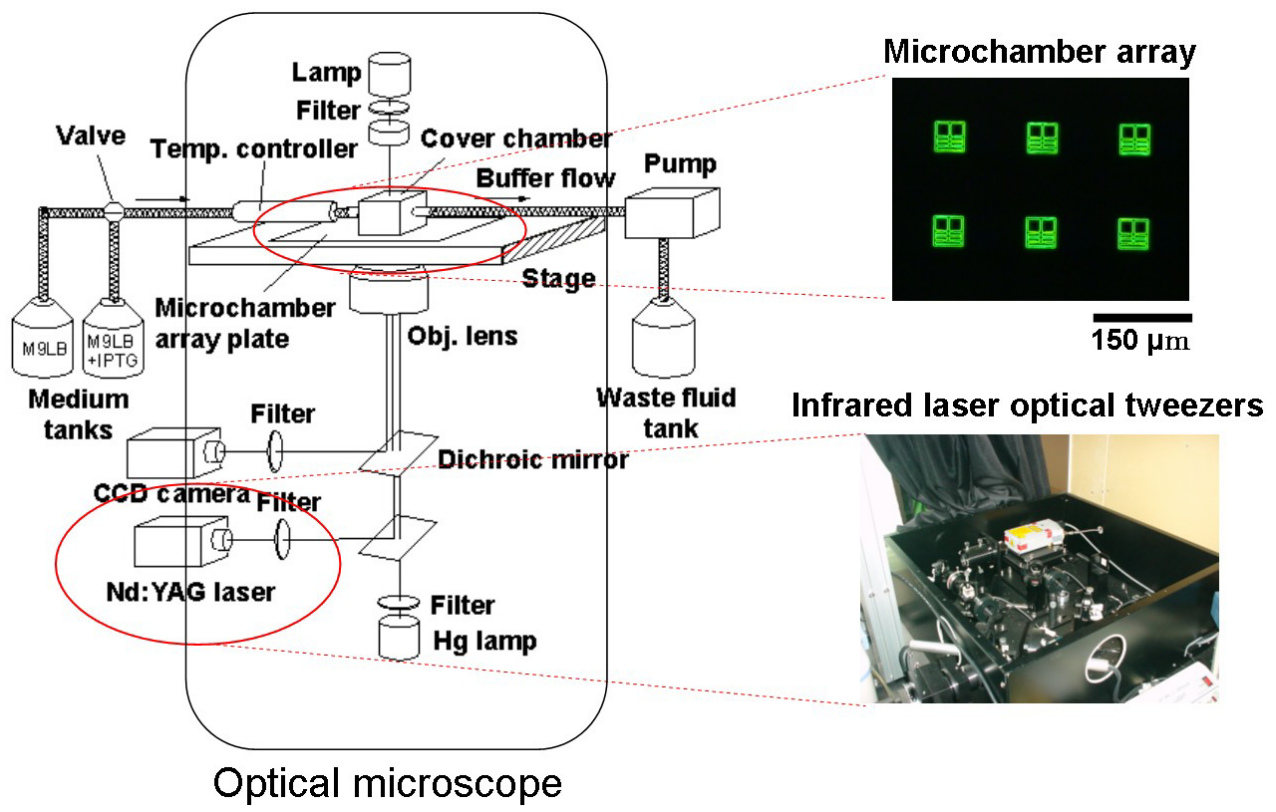
System for on-chip single-cell microculture chip.

To understand the variability of cells having the same genetic information and to observe the adaptation processes of cells, we need to compare the sister cells or the direct descendant cells directly (Fig. 3). For that purpose, we have developed the system for an on-chip single-cell cultivation chip. The system enables excess cells to be transferred from the analysis chamber to the waste chamber through a narrow channel and allows a particular cell to be selected from the cells in the microfabricated cultivation chamber by using a kind of non-contact force, optical tweezers (Fig. 5). Figure 6 depicts our entire system for the on-chip single-cell microculture chip. The system consists of a microchamber array plate, a cover chamber, a phase-contrast/fluorescent microscope and optical tweezers. The cover chamber is a glass cube filled with a buffer medium and is attached to the array plate so that the medium in the microchambers can be exchanged through a semipermeable membrane.

**Figure 7:**
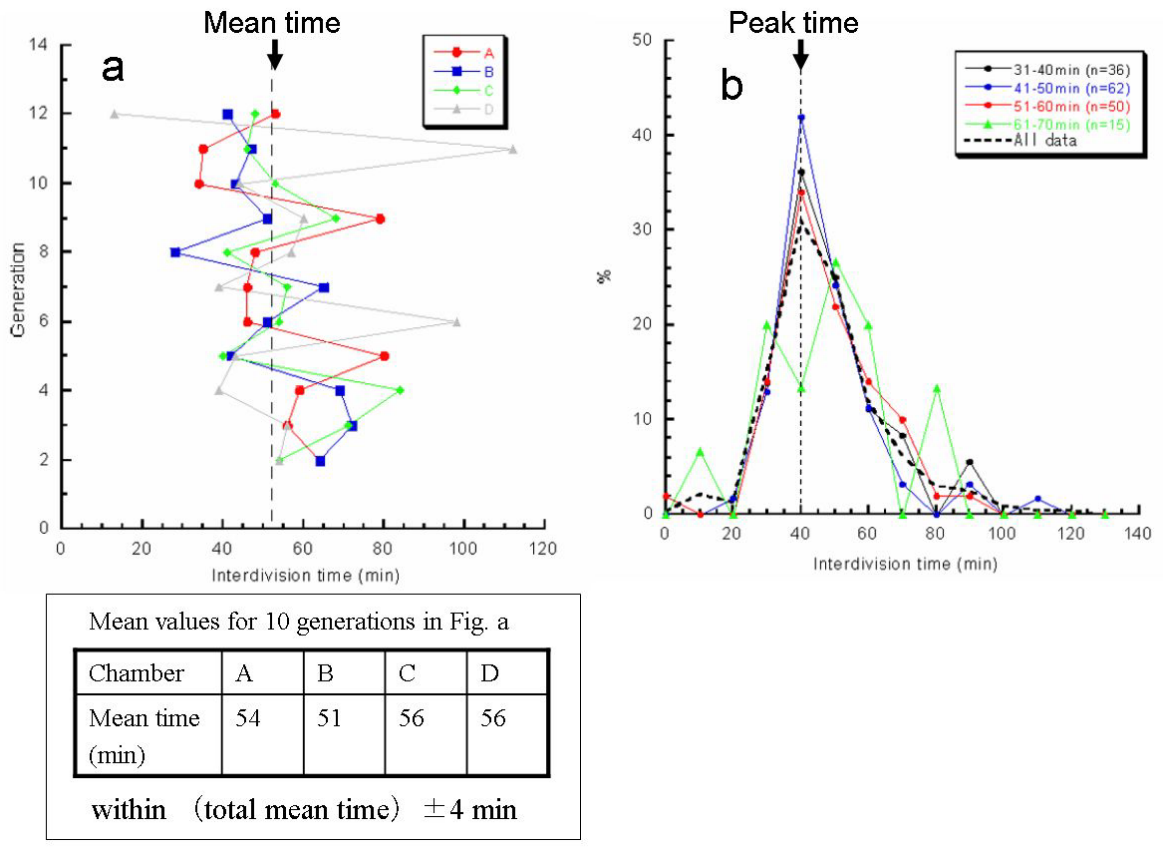
Genetic variability of direct descendant cells of *E. coli*.

Using the system, we examined whether the direct descendants of an isolated single cell could be observed under the same isolation conditions. Figure 7(a) plots the variations in interdivision times of consecutive generations of isolated *E. coli *cells derived from a common ancestor. The four series of interdivision times varied around the overall mean value, 52 min (dashed line); the mean values of the four cell lines a, b, c, and d were 54, 51, 56 and 56 min, showing differences rather small compared with the large variations in the interdivision times of consecutive generations. This result supports the idea that interdivision time variations from generation to generation are dominated by fluctuations around the mean value, and it was evidence of a stabilized phenotype that was subsequently inherited. To explore this idea, we examined the dependence of interdivision time on the interdivision time of the previous generation. We grouped the interdivision time data into four categories and determined their distributions (Fig. 7(b)). Comparison of these distributions showed that they were astonishingly similar to one other, suggesting that there was no dependence on the previous generation. That is, there was no inheritance in interdivision time from one generation to the next.

### 3-2. On-chip agarose microchamber system [11-14]

**Figure 8:**
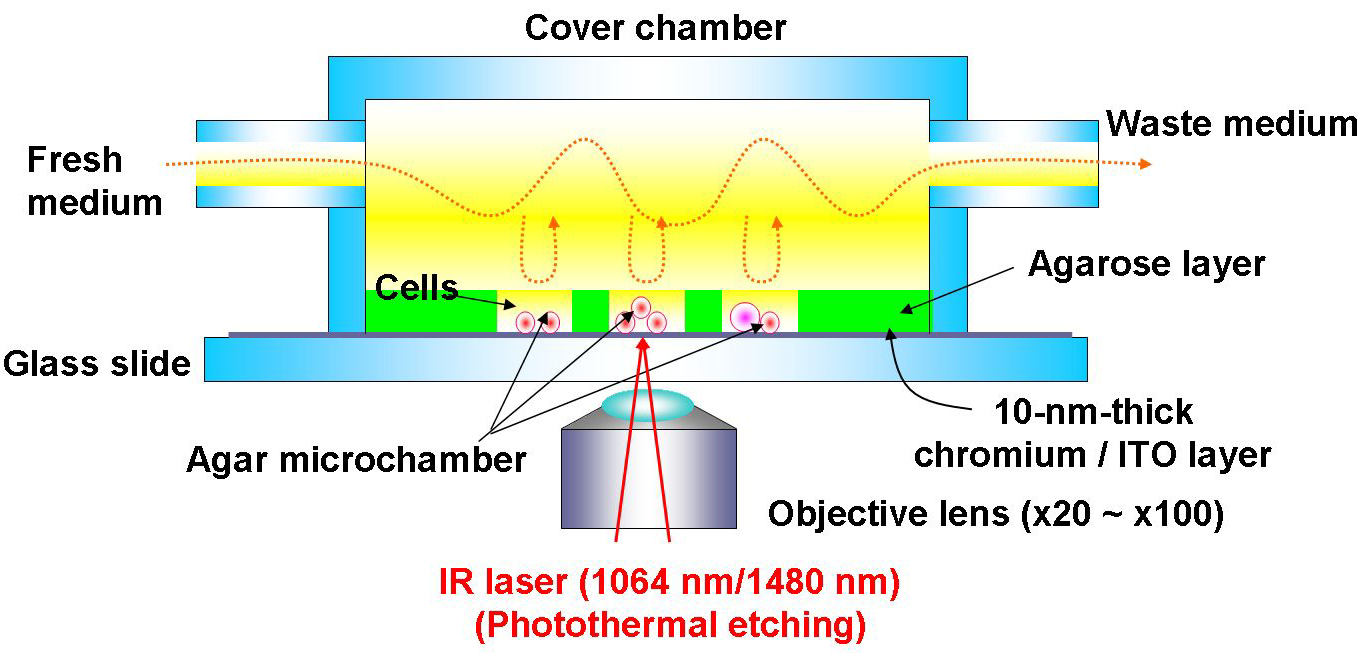
On-chip agarose microchamber system.

One approach to study network patterns (or cell-cell interactions) and the community effect of cells is to create a fully controlled network by using cells on the chip (Fig. 4). We have therefore developed a system consisting of an agar-microchamber (AMC) array chip, a cultivation dish with a nutrient-buffer-changing apparatus, a permeable cultivation container, and a phase-contrast/fluorescent optical microscope with a 1064-nm Nd:YAG focused laser irradiation apparatus for photothermal spot heating (Fig. 8). The most important advantage of this system is that we can change the microstructures in the agar layer even during cultivation, which is impossible when using conventional Si/glass-based microfabrication techniques and microprinting methods.

**Figure 9:**
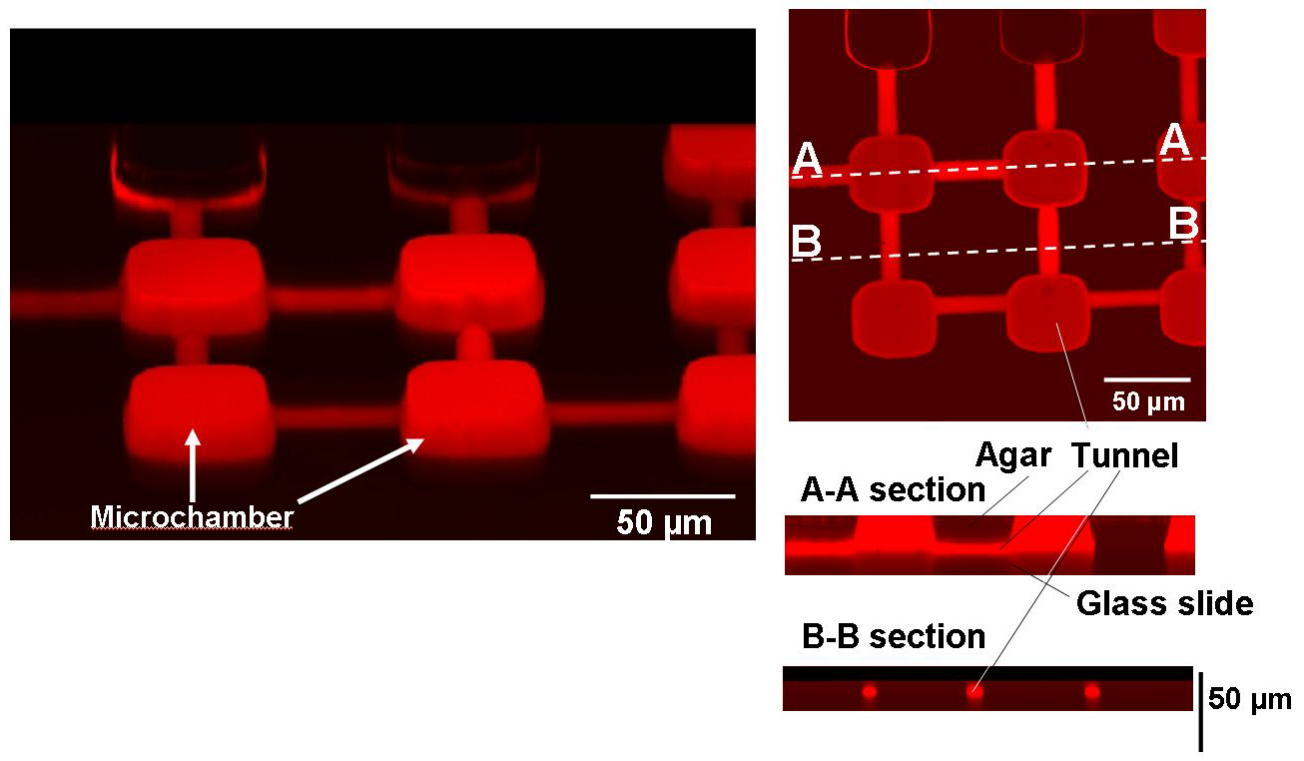
Three-dimensional structure of agarose microstructures.

As explained above, the agarose-microchamber cell-cultivation system includes an apparatus for photothermal etching. Photothermal etching is an area-specific melting of the agarose microchambers by spot heating using a focused laser beam and a thin layer made of a light-absorbing material such as chromium (since agarose itself has little absorbance at 1064-nm). We made the three-dimensional structure of the agar microchambers by using a photo-thermal etching module. Figure 9 is a top-view micrograph of the agar microchambers connected by small channels. The space on the chip was colored by filling the microchambers with a fluorescent dye solution. Also shown are cross-sectional views of the A-A and B-B sections, in which we can easily see narrow tunnels under the thick agar layer in the A-A section and round tunnels in the B-B section. These cross-sectional micrographs show that we can make narrow tunnels in the agar layer by photothermal etching. The left micrograph in Fig. 9 is a top view of the whole microchamber array connected by narrow tunnels.

**Figure 10:**
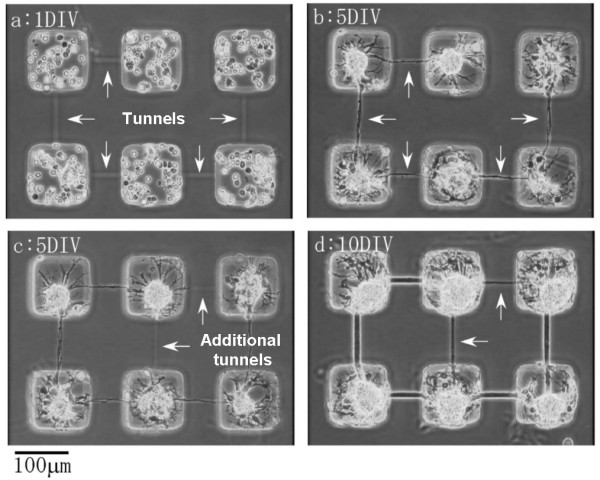
Stepwise formation of neuronal network of rat hippocampal cells.

By using this photothermal etching method, we can change the neural network pattern on a multi-electrode array chip during cultivation. Figure 10 shows the time course of the axon growth of rat hippocampal cells. After 5 days of cultivation (5DIV), when the cells in six microchambers had been connected by axons grown through the four existing tunnels (arrows in Figs. (a) and (b)), two new tunnels (arrows in Figs. (c) and (d)) were created by photothermal etching. After five more days of cultivation (10DIV), connecting axons had grown through them as well.

**Figure 11:**
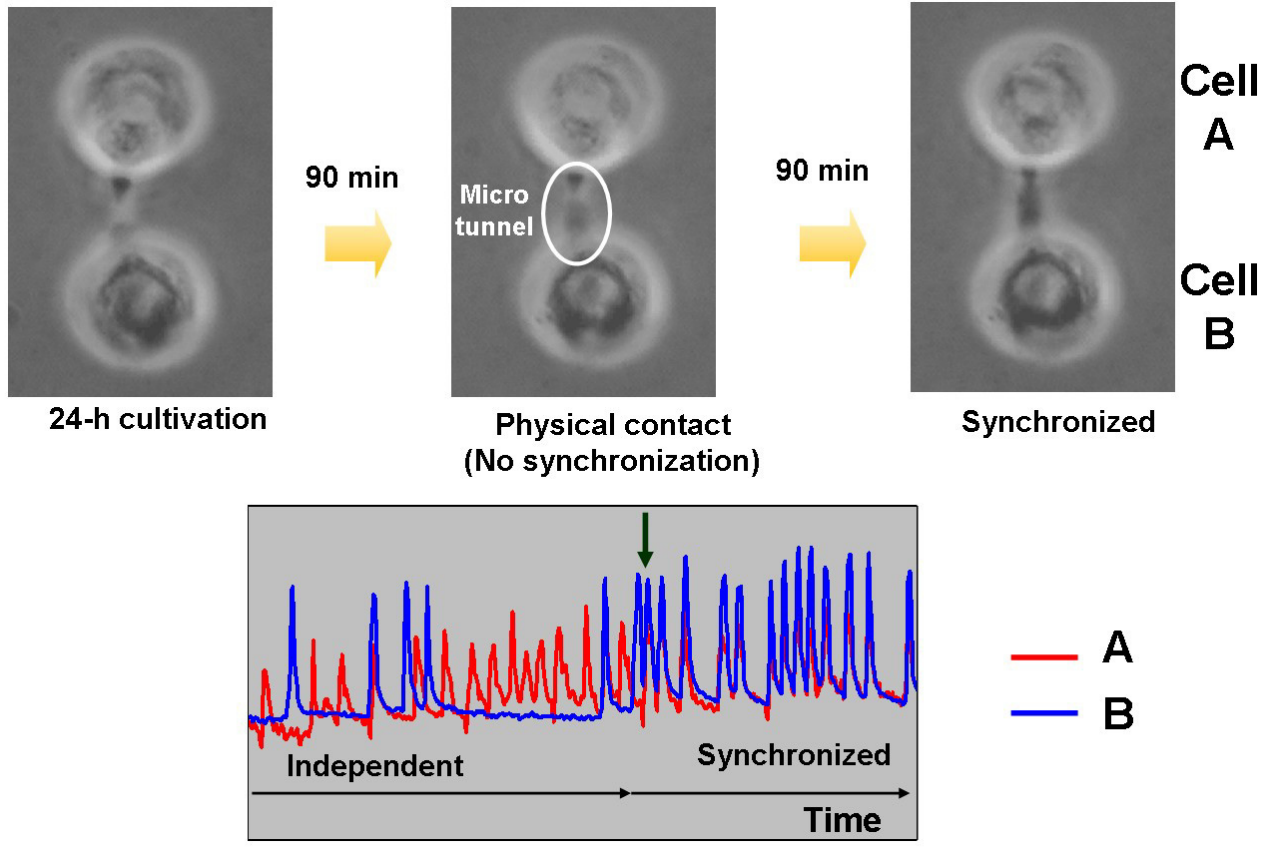
Dynamics of the synchronizing process of two isolated rat cardiac myocytes.

The agarose microchamber system can also be used to observe the dynamics of the synchronizing process of two isolated rat cardiac myocytes. Figure 11 shows an example of the synchronizing process of two cardiac myocytes. After the cultivation had begun, the two cells elongated and made physical contact within 24 hours, followed by synchronization. It should be noted that, as shown in the graph, the synchronization process involved one of the cells following the rhythm of the other, and that the 'copy cat' cell stops beating prior to acquiring the new beat rhythm.

## Conclusions

We have newly developed and have just started to use a series of methods for understanding the meaning of genetic information and epigenetic information in a simple cell model system. The most important expected contribution of this project is to reconstruct the concept of a cell regulatory network from the 'local' (molecules expressed at certain times and places) to the 'global' (the cell as a viable, functioning system). Knowledge of epigenetic information, which we can control and change during their life, is complementary to genetic information, and those two kinds of information are indispensable for living organisms. This new kind of knowlege has the potential to be the basis of a new field of science.

## Authors' contributions

KY conceived of the study, its design and coordination.
